# Global network random walk for predicting potential human lncRNA-disease associations

**DOI:** 10.1038/s41598-017-12763-z

**Published:** 2017-09-29

**Authors:** Changlong Gu, Bo Liao, Xiaoying Li, Lijun Cai, Zejun Li, Keqin Li, Jialiang Yang

**Affiliations:** 1 College of Information Science and Engineering, Hunan University, Changsha, Hunan 410082 China; 2 School of Computer and Information Science, Hunan Institute of Technology, Hengyang, 412002 China; 3 Department of Computer Science, State University of New York, New Paltz, New York, 12561 USA; 4 Department of Genetics and Genomic Sciences, Icahn School of Medicine at Mount Sinai, New York, 10029 USA

## Abstract

There is more and more evidence that the mutation and dysregulation of long non-coding RNA (lncRNA) are associated with numerous diseases, including cancers. However, experimental methods to identify associations between lncRNAs and diseases are expensive and time-consuming. Effective computational approaches to identify disease-related lncRNAs are in high demand; and would benefit the detection of lncRNA biomarkers for disease diagnosis, treatment, and prevention. In light of some limitations of existing computational methods, we developed a global network random walk model for predicting lncRNA-disease associations (GrwLDA) to reveal the potential associations between lncRNAs and diseases. GrwLDA is a universal network-based method and does not require negative samples. This method can be applied to a disease with no known associated lncRNA (isolated disease) and to lncRNA with no known associated disease (novel lncRNA). The leave-one-out cross validation (LOOCV) method was implemented to evaluate the predicted performance of GrwLDA. As a result, GrwLDA obtained reliable AUCs of 0.9449, 0.8562, and 0.8374 for overall, novel lncRNA and isolated disease prediction, respectively, significantly outperforming previous methods. Case studies of colon, gastric, and kidney cancers were also implemented, and the top 5 disease-lncRNA associations were reported for each disease. Interestingly, 13 (out of the 15) associations were confirmed by literature mining.

## Introduction

A non-coding RNA (ncRNA) is an RNA molecule that is not translated into protein. NcRNA was considered to be transcriptional noise for a long time. Recently, a large amount of evidence has indicated the key regulatory role of ncRNAs in numerous important biological processes^1^. According to their sizes, regulatory ncRNAs can be further classified as small and long ncRNAs^2^. Some ncRNAs, including miRNAs^3^, tRNAs^4^, and piRNAs^5^, have received attention from many researchers. Long ncRNAs (lncRNAs) are non-protein coding transcripts of a length greater than 200 nucleotides. In recent years, with the rapid development of experimental techniques and computational methods, an increasing number of lncRNAs have been discovered in eukaryotic organisms ranging from nematodes to humans. As of January 2016, 294 lncRNAs had been functionally annotated in the LncRNAdb database, which provides comprehensive annotations of eukaryotic lncRNAs^6,7^; 183 of these lncRNAs are annotated in humans.

Recently, the associations between lncRNAs and diseases have been widely studied. It is reported that mutations and dysregulations of lncRNAs are associated with a broad range of human diseases^8^, such as breast cancer^9^, colon cancer^10^, cardiovascular diseases^11^, and neurodegenerative diseases^12^. For example, lncRNA H19, BC200, and CDKN2B-AS1 have been experimentally confirmed to be closely related to breast cancer^13–15^. Therefore, identification of disease-related lncRNAs helps in understanding the molecular mechanism of diseases at the lncRNA level and further provides biomarkers for disease diagnosis, treatment, and prognosis. The lncRNA-disease associations have been increasingly reported in the past several years. By collecting and sorting lncRNA-related biological data from published studies, several researchers have established a few publicly available databases such as LncRNAdb^6^, LncRNADisease^16^, NRED^17^, and NONCODE^18^. These databases provide a fundamental basis for the study of lncRNAs, but only a small amount of lncRNA-disease associations were reported in these databases. Therefore, effective computational approaches to predict lncRNA-disease associations based on the datasets are in high demand.

Several powerful computational methods for predicting new human lncRNA-disease associations have been developed in recent years. According to their implementation strategy, these methods can be further classified as machine-learning-based methods and network-based methods.

The former class of computational methods predicts lncRNA-disease associations based on training datasets (i.e., known lncRNA-disease associations) and testing datasets (i.e., unknown lncRNA-disease associations)^8^. For example, Chen *et al*.^19^ developed LRLSLDA (laplacian regularized least squares for lncRNA-disease association) model to predict potential disease-related lncRNAs. LRLSLDA reveals the potential lncRNA-disease associations by integrating known lncRNA-disease associations with lncRNA expression profiles. LRLSLDA is a semi-supervised classification algorithm that does not require negative training samples. A major issue of LRLSLDA is how to select optimal parameters to obtain the best predicted performance. Subsequently, Chen *et al*.^20^ proposed a novel lncRNA similarity calculation method, namely, LNCSIM, and then used it to evaluate the predicted performance. LNCSIM showed a significant improvement for lncRNA-disease association prediction in a LOOCV process. By integrating genome, regulome, and transcriptome data, Zhao *et al*.^21^ proposed a naive Bayesian classifier to identify cancer-related lncRNAs. The results of ten-fold cross validation showed good performance, and 707 potential cancer-related lncRNAs were identified by this method. However, it is difficult to infer negative samples from this kind of method, which has become a key bottleneck to further research.

The latter class of computational methods predicts lncRNA-disease associations based on lncRNA similarities and disease similarities. The lncRNA and disease similarity network are connected by the known lncRNA-disease associations to form a heterogeneous network, based on the network to uncover the potential lncRNA-disease associations. A common assumption of this kind of method is that functionally similar lncRNAs tend to be associated with phenotypically similar diseases, and vice versa. Several researchers implemented random walks on heterogeneous networks to uncover the potential associations between lncRNAs and diseases^22–24^. For instance, Zhou *et al*.^24^ integrated a miRNA-associated lncRNA-lncRNA crosstalk network, disease-disease similarity network, and known lncRNA-disease association network into a heterogeneous network. Then a random walk was implemented with a restart on this heterogeneous network to prioritize candidate lncRNA–disease associations (RWRHLD). They used LOOCV to evaluate the predicted performance and obtained a reliable area under the curve (AUC) value of 0.871. RWRHLD implements a random walk from disease-related seed lncRNAs to other nodes; thus, this approach cannot be applied to predict isolated disease-related lncRNAs. By integrating a wide variety of biological data (disease semantic, lncRNA expression profiles, and known lncRNA-disease associations) into a heterogeneous network, Chen^25^ proposed the model of KATZ measure for lncRNA-disease association prediction (KATZLDA) to predict disease-related lncRNAs. Cross validation and case studies showed that KATZLDA offers good predicted performance. In particular, KATZLDA can predict isolated disease-related lncRNAs. However, the method relies excessively on a network topology structure; and may cause bias to diseases with more known related lncRNAs and lncRNAs with more known associated diseases.

In summary, existing computation methods for predicting lncRNA-disease associations have several limitations: (1) some approaches are unable to predict isolated disease-related lncRNAs; (2) some machine-learning-based methods require negative samples that are difficult to obtain; and (3) other approaches may be biased towards well-known lncRNAs and diseases. To overcome these limitations, we proposed a global network random walk for potential human lncRNA-disease association prediction (GrwLDA) to reveal the potential associations between lncRNAs and diseases. GrwLDA integrates disease semantic similarities, lncRNA functional similarities, and known lncRNA-disease associations to discover the potential associations.

The main contributions of the paper are summarized as follows.

1. GrwLDA integrates heterogeneous molecular data for inferring potential lncRNA-disease associations.
2. GrwLDA is a universal network-based method and does not require negative samples.
3. GrwLDA can be applied to predict isolated disease (i.e., disease without any known related lncRNA), related lncRNAs, and novel lncRNA- associated diseases (i.e., lncRNA without any known associated disease).

## Results

### Performance evaluation

LOOCV was implemented on the benchmark dataset to evaluate the predicted performance of GrwLDA and two state-of-the-art computational models: LRLSLDA^19^ and KATZLDA^25^.

One lncRNA-disease association was excluded (set to 0), and the predictor score was recovered by remaining associations. All predictor scores were sorted, and a special ranking position was selected as a threshold. True positives (*TP*) were the number of the known associations above the threshold, whereas false positives (*FP*) were the number of the unknown associations above the threshold. True negatives (*TN*) were the number of the unknown associations below the threshold, whereas false negatives (*FN*) were the number of the known associations below the threshold. The receiver operating characteristic (ROC) curve plotted the test sensitivity or true-positive rate $(TPR=\frac{TP}{TP+FN})$ versus 1-specificity or false-positive rate $(FPR=\frac{FP}{FP+TN})$ at different thresholds and the precision-recall (PR) curve plotted precision $(precision=\frac{TP}{TP+FP})$ versus recall $(recall=\frac{TP}{TP+FN})$ at different thresholds. Specifically, the area under the ROC curve (AUC) and the area under the PR curve (AUPR) were adopted to evaluate the performances.

**Figure 1:**
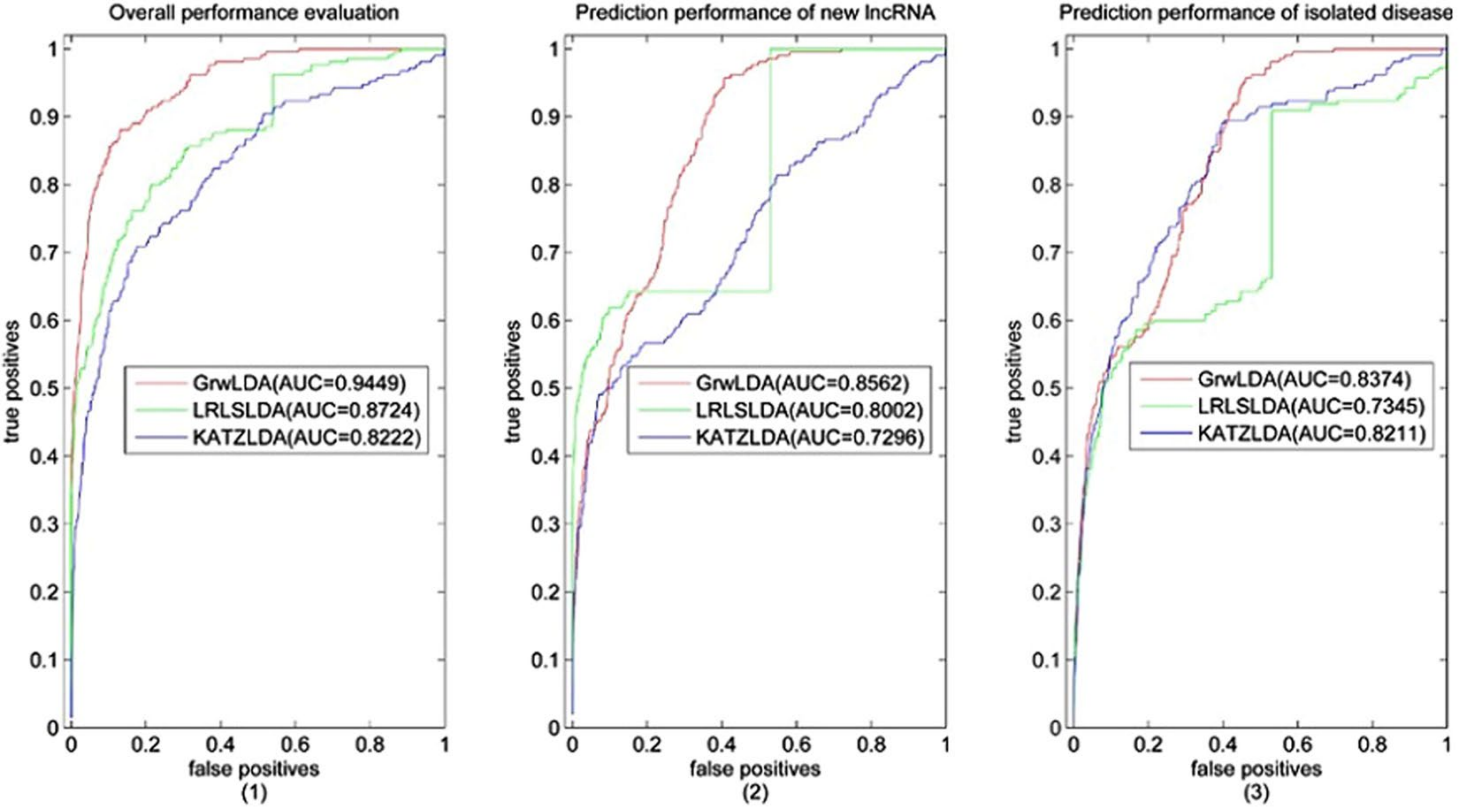
Performance comparisons of GrwLDA, LRLSLDA and KATZLDA in terms of ROC curves and AUCs based on LOOCV. (1) The overall predicted performance evaluation; (2) The predicted performance of novel lncRNA-associated diseases prediction; (3) The predicted performance of isolated disease-related lncRNAs prediction.

**Figure 2:**
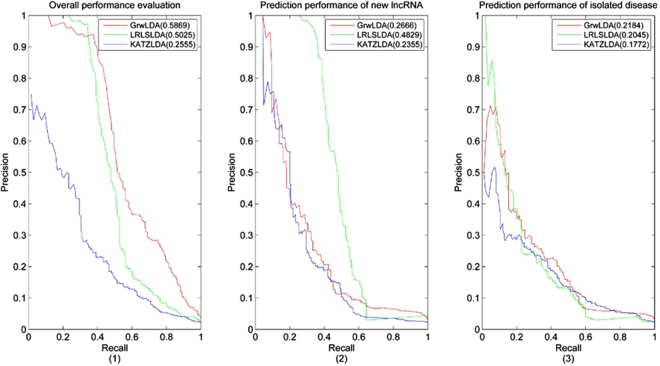
Performance comparisons of GrwLDA, LRLSLDA and KATZLDA in terms of PR curves and AUPRs based on LOOCV. (1) The overall predicted performance evaluation; (2) The predicted performance of novel lncRNA-associated diseases prediction; (3) The predicted performance of isolated disease-related lncRNAs prediction.

The three approaches can reconstruct missing associations for all the diseases simultaneously; and can predict potential lncRNA-disease associations for novel lncRNA and isolated disease. To comprehensively compare the predicted performance of the above three methods, we implement LOOCV on the benchmark dataset while considering the following aspects: (1) the overall performance evaluation; (2) the predicted performance of novel lncRNA-associated diseases prediction (when calculating the predictor score between lncRNA *i* and disease *j*; all associations between lncRNA *i* and all diseases are excluded and its score is recovered by remaining associations); and (3) the predicted performance of isolated disease-related lncRNAs prediction (all associations among all lncRNAs and disease *j* are excluded, and the predictor score between lncRNA *i* and disease *j* is recovered by the remaining associations). Four parameters, namely, *γ*, the restart probability of RWR, the two balance parameters *α* and *β*, and the integrate parameter *η* were employed in our model. We obtained the optimal parameters by experiments. We implemented the LOOCV of GrwLDA method by setting the four parameters from 0.1 to 0.9; the optimal parameters are *γ* = 0.9, *α* = 0.1, *β* = 0.1, and *η* = 0.7. The optimal parameters were selected for LRLSLDA and KATZLDA as described in the literature. The ROC curves and PR curves of the previously mentioned features were plotted and are shown in Fig. 1 and Fig. 2, respectively, and the AUC values and AUPR values are shown in their legends.

As seen from the figures, for the overall performance evaluation, GrwLDA has an AUC of 0.9449 and AUPR of 0.5869, which are better than those of LRLSLDA and KATZLDA; and for the predicted performance of isolated disease-related lncRNAs prediction, GrwLDA has an AUC of 0.8374 and AUPR of 0.2184, also ahead of those of LRLSLDA and KATZLDA. Although the LRLSLDA method obtained the best AUPR value of novel lncRNA-associated diseases prediction, its AUC value was significantly lower than that of GrwLDA.

The comparison among the above three methods based on 5-fold cross validation was implemented to further demonstrate the predictive ability of GrwLDA. The benchmark dataset was randomly divided into five parts, one for testing and the rest as a training set. In other words, all associations in the testing set were removed, and their predictor scores were regenerated by other associations. After all the predictor scores were obtained, the ROC curve was drawn, and the AUC value was calculated. The 5-fold cross validation was performed 10 times, and the average AUC value was adopted to evaluate the performances. As a result, GrwLDA had an average AUC of 0.9201, and those of LRLSLDA and KATZLDA were 0.8585 and 0.8145, respectively.

In conclusion, GrwLDA demonstrated significant performance improvements over previous computational models in the evaluation framework of LOOCV and 5-fold cross validation.

### Case study

Evidence from a wide range of sources suggests that lncRNAs play critical roles in the development of various cancers. To further evaluate the performance of GrwLDA in predicting potential disease-related lncRNAs, colon cancer, kidney cancer and gastric cancer were chosen as case studies. All known associations were used as the training set, and the unknown associations were assigned as the testing set. Then, the unknown lncRNA-disease associations of each disease were ranked according to the predicted results of GrwLDA, and the top five were selected for further validation. The predicted results were verified based on newly updated disease-lncRNA associations in the LncRNADisease database and in a few recently published studies. The predicted results and verified evidence are listed in Table 1.

**Table 1:** The top five predicted results for colon cancer, kidney cancer and gastric cancer. Only two associations are not confirmed by the latest research literature.

rank	disease	lncRNA	evidence
1	Colon cancer	HOTAIR	LncRNADisease
2	Colon cancer	MALAT1	LncRNADisease
3	Colon cancer	CRNDE	LncRNADisease
4	Colon cancer	PVT1	literature^26^
5	Colon cancer	KCNQ1OT1	unconfirmed
1	Kidney cancer	H19	LncRNADisease
2	Kidney cancer	GNAS-AS1	unconfirmed
3	Kidney cancer	PVT1	LncRNADisease
4	Kidney cancer	WT1-AS	literature^29^
5	Kidney cancer	KCNQ1DN	literature^30^
1	Gastric cancer	H19	LncRNADisease
2	Gastric cancer	HOTAIR	LncRNADisease
3	Gastric cancer	MEG3	LncRNADisease
4	Gastric cancer	PVT1	LncRNADisease
5	Gastric cancer	MALAT1	literature^31^

Colon cancer is one of the most common malignant tumors worldwide, killing almost 700,000 people every year. This cancer is a disease of modernity, with the highest rates of incidence being recorded in developed countries. Biological experiments have demonstrated several important associations between colon cancer and the dysregulation of lncRNAs. The potential colon cancer-related lncRNAs were predicted by GrwLDA. As a result, the associations between colon cancer and HOTAIR, CRNDE, and MALAT1 (top 3 predictions) were verified by the updates in the LncRNADisease database. Furthermore, Tseng *et al*.^26^ showed that ablation of PVT1 (ranked fourth) from the MYC-driven colon cancer line HCT116 diminishes tumorigenic potency. Although there is no direct evidence validating that KCNQ1OT1 is associated with colon cancer, it has been considered as an effective biomarker for disease diagnosis^27^ due to the high frequency of the loss of KCNQ1OT1imprinting in colon cancer.

Kidney cancer, also known as renal cancer, is a disease that starts in the kidneys. Kidney cancer occurs when healthy cells in one or both kidneys grow out of control and form a lump (called a tumor). Kidney cancer is the 12^th^ most common cancer worldwide, with more incidences in men than women; in addition, it is more prevalent in developed countries, with the highest rates being observed in North America and Europe, while the lowest are found in Africa and Asia^28^. GrwLDA was implemented to identify kidney cancer-related lncRNAs. The predicted kidney-related lncRNAs, H19 and PVT1 (ranked first and third in the predicted results, respectively) have already been validated according to the LncRNADisease database. Furthermore, Dallosso *et al*.^29^ and Xin *et al*.^30^ respectively inferred that WT1-AS (ranked 4^th^) and KCNQ1DN (ranked 5^th^) are associated with Wilms’ tumor, a cancer of the kidneys that usually affects newborns and the very young.

Gastric cancer is the third most common cause of cancer-related deaths in the world. This type of cancer remains difficult to cure in Western countries, primarily because most patients present with an advanced disease stage. Therefore, the identification of novel molecules associated with gastric cancer is beneficial to the diagnosis and treatment of gastric cancer. We also implemented GrwLDA to identify potential gastric cancer-related lncRNAs. The top 4 predicted gastric cancer-related lncRNAs, H19, HOTAIR, MEG3, and PVT1 were confirmed by the updates of the LncRNADisease database. In addition, Wang *et al*.^31^ reported that MALAT1 (ranked 5^th^) promotes cell proliferation in gastric cancer by recruiting SF2/ASF.

To further compare the predicted performance of GrwLDA, LRLSLDA, and KATZLDA, we curated a list of newly collected lncRNAs associated with colon, gastric and kidney cancers by the updates of the LncRNADisease database, which were considered as the ground truth; we then ranked three methods based on the list (Table 2). Finally, we calculated the average ranking of the three diseases together. With an average ranking of 19 distinct, experimentally confirmed lncRNA-disease associations for these three important diseases, GrwLDA outperformed LRLSLDA and KATZLDA.

**Table 2:** Performance comparisons of GrwLDA, LRLSLDA and KATZLDA methods based on the newly collected lncRNAs associated with colon, gastric and kidney cancer by the updates of LncRNADisease database and their ranking of the three methods.

disease	lncRNA	LRLSLDA	KATZLDA	GrwLDA
Colon cancer	HOTAIR	2	2	1
Colon cancer	CRNDE	19	31	3
Colon cancer	MALAT1	1	1	2
Colon cancer	KCNQ1OT1	8	23	5
Colon cancer	LSINCT5	21	11	15
Gastric cancer	H19	2	1	1
Gastric cancer	HOTAIR	1	5	2
Gastric cancer	MEG3	3	2	3
Gastric cancer	PVT1	4	3	4
Gastric cancer	CDKN2B-AS1	7	6	7
Gastric cancer	LSINCT5	14	15	23
Gastric cancer	UCA1	70	19	16
Gastric cancer	SPRY4-IT1	72	44	45
Kidney cancer	H19	6	1	1
Kidney cancer	PVT1	12	3	3
Kidney cancer	MEG3	15	2	7
Kidney cancer	MALAT1	26	4	10
Kidney cancer	GAS5	45	15	29
Kidney cancer	KCNQ1OT1	66	36	37
Average ranking of the three diseases		20.74	11.79	11.26

### Application of GrwLDA to predict isolated disease-related lncRNAs and novel lncRNA-associated diseases

Research into lncRNA is still in its infancy, and numerous diseases associated with lncRNAs have yet to be confirmed. Therefore, the prediction and identification of isolated disease-related lncRNAs has become an important task in lncRNA research. GrwLDA was implemented to predict isolated disease-related lncRNAs. We removed the known and verified lncRNA-disease associations related to predictive diseases. This operation ensures that we only use similarity information and known lncRNA-disease associations of the other diseases to predict disease-related lncRNAs. Isolated disease-related lncRNAs prediction was implemented for colon, kidney and gastric cancers, and the top five predicted results of each disease were listed in Table 3. As a result, 13 of the 15 predicted results were confirmed by the updates of the LncRNADisease database and by some recent literatures.

**Table 3:** Isolated disease-related lncRNA prediction was implemented for colon, kidney and gastric cancers; the top five predicted results of each disease are listed. A total of 13 of the 15 predicted results are confirmed by the updates of the LncRNADisease database and by the latest research literature.

rank	disease	lncRNA	evidence
1	Colon cancer	HOTAIR	LncRNADisease
2	Colon cancer	PVT1	literature^26^
3	Colon cancer	MALAT1	LncRNADisease
4	Colon cancer	CRNDE	LncRNADisease
5	Colon cancer	KCNQ1OT1	unconfirmed
1	Kidney cancer	H19	LncRNADisease
2	Kidney cancer	PVT1	LncRNADisease
3	Kidney cancer	MEG3	LncRNADisease
4	Kidney cancer	MALAT1	LncRNADisease
5	Kidney cancer	GNAS-AS1	unconfirmed
1	Gastric cancer	H19	LncRNADisease
2	Gastric cancer	HOTAIR	LncRNADisease
3	Gastric cancer	MEG3	LncRNADisease
4	Gastric cancer	PVT1	LncRNADisease
5	Gastric cancer	MALAT1	literature^31^

Novel lncRNAs are a class of lncRNAs that target unavailable disease association information. To verify that our method is able to prioritize diseases for novel lncRNAs, we removed all experimentally verified associations related to lncRNA. This step ensured that only similarity information and known lncRNA-disease associations of the other lncRNAs were used to predict potential associations. GrwLDA was also implemented to predict novel lncRNA-associated diseases. Novel lncRNA-associated disease prediction was implemented for H19, HOTAIR, and MALAT1; the top five of each lncRNA predicted results are listed in Table 4. As a result, 14 of the 15 predicted results are confirmed by the updates of the LncRNADisease database and a few recent journal articles.

**Table 4:** Novel lncRNA-associated diseases predicting H19, HOTAIR and MALAT1 and the top five of each lncRNA-predicted results are listed. As a result, 14 of the 15 predicted results are confirmed by the updates of the LncRNADisease database and by the latest research literature.

rank	lncRNA	disease	evidence
1	H19	Prostatic Neoplasms	LncRNADisease
2	H19	Lymphoma	literature^37^
3	H19	Colorectal Neoplasms	LncRNADisease
4	H19	Testicular Neoplasms	literature^38^
5	H19	Neuroblastoma	LncRNADisease
1	HOTAIR	Prostatic Neoplasms	literature^39^
2	HOTAIR	Lymphoma	literature^40^
3	HOTAIR	Ovarian Neoplasms	literature^41^
4	HOTAIR	Testicular Neoplasms	unconfirmed
5	HOTAIR	Melanoma	literature^42^
1	MALAT1	Breast Neoplasms	LncRNADisease
2	MALAT1	Prostatic Neoplasms	literature^39^
3	MALAT1	Lymphoma	literature^43^
4	MALAT1	Ovarian Neoplasms	literature^44^
5	MALAT1	Melanoma	literature^45^

In conclusion, GrwLDA exhibits good performance in inferring isolated disease-related lncRNAs and novel lncRNA-associated diseases.

## Discussion

Accumulating evidence has indicated that lncRNAs play important roles in the development of diseases. Identification of disease-related lncRNAs will be beneficial to gain a deeper understanding of disease mechanisms at the molecular level. As valuable complements to experimental studies, computational models used to identify associations between lncRNAs and diseases are in high demand.

In this article, by integrating known lncRNA-disease associations, disease semantic similarities, and lncRNA functional similarities, a method called GrwLDA was developed to predict potential lncRNA-disease associations on a large scale. GrwLDA is a universal network-based method can be applied to predict isolated diseases and novel lncRNAs without any known associations. GrwLDA achieved LOOCV AUCs of 0.9449, 0.8562, and 0.8374 for overall, novel lncRNA and isolated disease prediction, respectively, which were considerably higher values than those obtained by existing computational models. Furthermore, by applying the GrwLDA method to colon, gastric, and kidney cancer as case studies, 13 potential associations in the top five predictions for these important diseases were confirmed by recent studies.

Despite the favorable results obtained using GrwLDA, this study presents certain limitations. First, given that available experimentally validated lncRNA-disease associations are still relatively rare and the lncRNA similarities are calculated based on them, GrwLDA will probably produce biased predictions. This problem is common to predicting lncRNA-disease associations. With the development of lncRNA-related research, more comprehensive data will be obtained and will improve the prediction performance of the GrwLDA method. Second, more reliable information sources, such as lncRNA-miRNA interactions and lncRNA expression profiles can be integrated to measure the lncRNA functional similarities. Third, parameter selection of GrwLDA is difficult, and we selected the optimal parameters by experience. Therefore, the parameter optimization method for GrwLDA should be studied in the future. Finally, GrwLDA implements random walk with restart from lncRNA seed nodes and disease seed nodes, which will result in two stable transition probabilities. Researching how to obtain the final score with a single measurement or a more reliable integration method should be prioritized in future studies.

## Materials and Methods

### LncRNA-disease associations

The known human lncRNA-disease association dataset is downloaded from LncRNADisease database (http://www.cuilab.cn/lncrnadisease) in October 2012. The data preprocessing process is as follows. (1) The diseases of the dataset are mapped to MeSH description by the MeSH database (https://www.ncbi.nlm.nih.gov/mesh). (2) The repeated associations and several diseases without any MeSH descriptors or tree numbers are removed. (3) All data are screened through homo sapiens. (4) The classification of the gene sequence is determined by querying the Nucleotide database (https://www.ncbi.nlm.nih.gov/nuccore); if the gene class is not lncRNA, such as 7SK (class is snRNA) and 7SL (class is scRNA), the gene will be removed. After pretreatment, 210 distinct high-quality experimental verified lncRNA-disease associations are obtained, including 78 lncRNAs and 113 diseases. We use this dataset as the benchmark dataset and variables *nl* and *nd* to represent the number of lncRNAs and diseases, respectively. We let matrix *AS* denote the adjacency matrix of lncRNA-disease associations, where the entity *AS*(*i*, *j*) in row *i* and column *j* is 1 if lncRNA *i* is associated with disease *j*, otherwise 0.

### Disease semantic similarities

According to the disease tree numbers and disease semantic terms, each disease can be described as a directed acyclic graph (DAG). The DAG *G*(*D*) = (*V*(*D*), *E*(*D*)) is used to present disease *D*, where *V*(*D*) is the vertex set including the disease *D* and its ancestor nodes, and *E*(*D*) is the set of connecting edges including the direct edges from parent nodes to child nodes. In the same manner as described in the literature^32^, the contribution of each disease semantic term of disease *D* is numerically investigated as follows:

1 $$\{\begin{array}{c}D{T}_{D}(D)=1\\ D{T}_{D}(t)=\,\max \,\{{\rm{\Delta }}\times D{T}_{D}({t}^{\text{'}})|{t}^{\text{'}}\in children\,of\,t\}\,if\,t\ne D\end{array}$$

where ${\rm{\Delta }}\in [0,1]$ is the semantic contribution decay factor. In the DAG of disease *D*, disease *D* is the most specific disease, and its contribution to its own semantic score is defined 1. The disease term located far from disease *D* is considered to be a more general disease, and its contribution is multiplied by the semantic contribution decay factor. The semantic score of disease *D* is defined in Equation (2):

2 $$T(D)=\sum _{t\in V(D)}D{T}_{D}(t)$$

on the basis of the shared nodes in two disease DAGs, we calculate disease semantic similarity between disease *A* and disease *B* defined as Equation (3):

3 $$DD(A,B)=\frac{{\sum }_{t\in (V(A){\cap }^{}V(B)}(D{T}_{A}(t)+D{T}_{B}(t))}{DT(A)+DT(B)}$$

where *DD* is the disease semantic similarity matrix and *DD*(*i*, *j*) in row *i*, and column *j* represents the semantic similarity between diseases *i* and *j*. The disease semantic similarity between diseases *i* and *j* is measured based on both the addresses of these diseases in DAGs and their semantic relations with their ancestor diseases.

### LncRNA functional similarities

The lncRNA functional similarities are calculated using LNCSIM model^20^. The LNCSIM model quantitatively calculates the functional similarities between two lnRNAs by measuring the semantic similarity between the two lncRNA-related disease groups. LNCSIM defines *D*(*u*) and *D*(*v*) as the disease groups associated with lncRNAs *u* and *v*, respectively; and calculates the similarity between *D*(*u*) and *D*(*v*) as the functional similarity of lncRNAs *u* and *v*. LNCSIM first calculates the similarity between one disease and a disease group. For example, the similarity between disease *d*1 (a member of *D*(*u*)) and disease group *D*(*v*) was calculated as follows:

4 $$S(d1,D(v))=\mathop{\max }\limits_{d\in D(v)}(DD(d1,d)$$

and the similarity between lncRNA *u* and *v* was defined as Equation (5):

5 $$LL(u,v)=\frac{{\sum }_{d\in D(u)}S(d,D(v)+{\sum }_{d\in D(v)}S(d,D(u)}{|D(u)|+|D(v)|}$$

where $|D(u)|$ and $\,|D(v)|$ are the numbers of diseases associated with lncRNAs *u* and *v*, respectively. We use matrix *LL* to denote the lncRNA functional similarities, where the variable *LL*(*i*, *j*) in row *i* and column *j* is the functional similarity between lncRNA *i* and lncRNA *j*. Based on the common assumption that the more similar the two lncRNA associated diseases are, then the more similar their functions are, Equation (5) calculates the functional similarity of two lncRNAs based on their respective associated disease group.

### Constructing probability transfer matrix

We define Equation (6) thereby forming a probability transfer matrix to normalize each of the columns of matrix *M*_*m* × *n*_.

6 $$WM(i,j)=\{\,\begin{array}{cc}\frac{M(i,j)}{{\sum }_{k=1}^{m}M(k,j)} & \,if\,\sum _{k=1}^{m}M(k,j)\ne 0\\ 0 & \,if\,\sum _{k=1}^{m}M(k,j)=0\end{array}$$

The matrices *LL*, *DD*, *AS*, and *AS* ^*T*^ (transpose matrix of *AS*) are normalized by Equation (6), and we obtain the normalized matrices *WL*, *WD*, *WA*1, and *WA*2, respectively.

### Global network random walk model for predicting potential human lncRNA-disease (GrwLDA)

**Figure 3:**
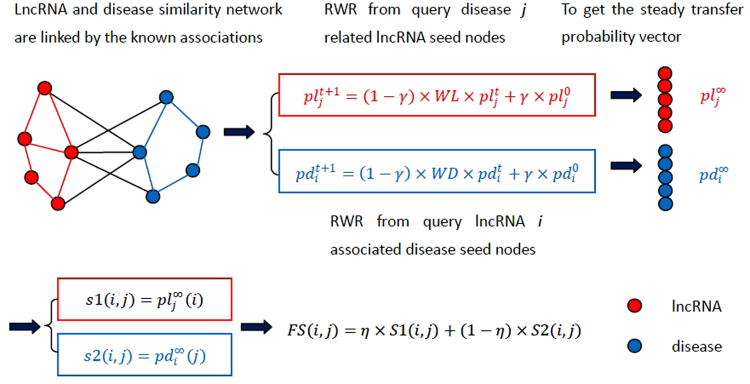
Flowchart of GrwLDA. The GrwLDA method is implemented in three steps as follows: (1) RWR is restarted from lncRNA seed nodes associated with query disease; (2) RWR is restarted from disease seed nodes associated with query lncRNA; and (3) the potential lncRNA-disease associations are predicted by integrating the results of step (1) and step (2).

The flowchart of the GrwLDA method is shown in Fig. 3.

Random walk with restart (RWR) algorithms are derived from graph theory and randomly simulates a random walker’s transition from its current nodes to its neighbors in the network starting at several given seed nodes. Many researchers have successfully applied the RWR algorithm in their specific application^33–36^. For instance, Sebastian *et al*.^33^ implemented the RWR algorithm on a global protein-protein interaction (PPI) network for prioritizing candidate disease genes; and focused on the functional link between miRNA targets and disease genes in a PPI network. Shi *et al*.^34^ used the RWR algorithm for predicting potential miRNA-disease associations. In this work, inspired by previous studies, we propose a global network random walk model for predicting potential human lncRNA-disease. The GrwLDA method is implemented in three steps, as follows: (1) RWR is restarted from lncRNA seed nodes associated with query disease. (2) RWR is restarted from disease seed nodes associated with query lncRNA. (3) The potential lncRNA-disease associations are predicted by integrating results of step (1) and step (2).

The detailed implementation procedure of the GrwLDA method to calculate the predictor score between lncRNA *i* and disease *j* is as follows.

First, based on the common assumption that lncRNAs with similar functions are normally associated with phenotypically similar diseases and vice versa, the GrwLDA method implements RWR from lncRNA seed nodes associated with disease *j*, and the transition probability from lncRNA *i* to disease *j* is obtained. We let $p{l}_{j}^{0}$ be the initial probability vector of disease *j* and $p{l}_{j}^{t}$ be a vector consisting of the transition probability from all lncRNAs to disease *j* at step *t*. Therefore, the probability vector at step *t* + 1 can be iteratively calculated by Equation (7):

7 $$p{l}_{j}^{t+1}=(1-\gamma )\times WL\times p{l}_{j}^{t}+\gamma \times p{l}_{j}^{0}$$

where $\gamma \,{\epsilon }\,(0,1)$ indicates the restart probability, and *WL* is the probabilistic weight network of lncRNAs. To make full use of global network similarity information, the global relevance score between disease *j* and all diseases is approximately calculated as follows:

8 $$LPd(j)=(1-\alpha )\times (I-\alpha \times WD)\times d(j)\,$$

where *d*(*j*) is a binary column vector of length *nd*, with a *jth* element of 1 and other elements being 0. Vector *LPd*(*j*) is the Laplacian score vector of query disease *j*, and *α* ∈ (0, 1) is a balance parameter. To force connected diseases to receive similar scores and ensure the consistency with the query disease, the Laplacian score vector of query disease *j* is smoothed by parameter α. Unlike traditional RWR, we construct the initial probability vector of disease *j* considering the associated lncRNA seed nodes and the Laplacian score vector of query disease *j* simultaneously:

9 $$p{l}_{j}^{0}=\,WA1\times LPd(j)+WA1(:,j)$$

and then normalize by Equation (6). Then, the transition probability of initial seed nodes of disease *j* is acquired. We then implement RWR by Equation (7), and after several steps, the steady probability $p{l}_{j}^{\infty }$ is obtained when the change between $p{l}_{j}^{t+1}$ and $p{l}_{j}^{t}$ is less than 10^−6^, and the transition probability from lncRNA *i* to disease *j* is obtained using Equation (10):

10 $$s1(i,j)=p{l}_{j}^{\infty }(i)$$

According to Equation (7), random walk from the disease *j*-related lncRNA seed nodes, for any node, will be probabilistic 1 − *γ* transferred to its neighbor nodes and probabilistic *γ* back to the seed nodes. The greater the similarity between the nodes is, the greater the transition probability will be. At the end of the iteration, *s*1(*i*, *j*) is the probability of lncRNA *i* to disease *j*, and the greater the value is, the greater is the likelihood of the association.

Second, based on the assumption that phenotypically similar diseases are normally associated with functional similarity lncRNAs and vice versa, the GrwLDA method implements RWR from disease seed nodes associated with lncRNA *i* to obtain the transition probability from disease *j* to lncRNA *i*. Similar to the first step, graph Laplacian scores can be derived to measure the global relevance between lncRNA *i* and all lncRNAs as follows:

11 $$\,LPl(i)=(1-\beta )\times (I-\beta \times WL)\times l(i)$$

where *l*(*i*) is a binary column vector of length *nl*, with an *ith* element of 1 and other elements being 0. Vector *LPl*(*i*) is the Laplacian score vector of query lncRNA *i*, and *β* ∈ (0, 1) is a balance parameter. To focus on lncRNA-disease associations and the global lncRNA-lncRNA similarities simultaneously, we defined the initial transition probability from lncRNA *i* to all diseases as:

12 $$\,p{d}_{i}^{0}=WA2\times LPl(i)+WA2(:,i)$$

and then we implemented RWR from disease seed nodes associated with lncRNA *i* as follows:

13 $$\,\,p{d}_{i}^{t+1}=(1-\gamma )\times WD\times p{d}_{i}^{t}+\gamma \times p{d}_{i}^{0}$$

where$\,\,\,p{d}_{i}^{t+1}$,$\,\,p{d}_{i}^{t}$ and$\,\,p{d}_{i}^{0}$ are the transition probability vector from lncRNA *i* to all disease nodes at (*t* + 1), (*t*)*th* and (0)*th* step of iteration, separately. After several steps, the steady probability $p{d}_{i}^{\infty }$ and the transition probability from lncRNA *i* to disease *j* were obtained as:

14 $$s2(i,j)=p{d}_{i}^{\infty }(j)$$

For similar reasons, the greater the value of $s2(i,j)$ is, the greater the likelihood that lncRNA *i* and disease *j* are associated.

Finally, the transition probability from lncRNA *i* to disease *j* obtained from the previous two steps is integrated as the final score (*FS*) to predict the potential lncRNA-disease associations:

15 $$FS(i,j)=\eta \times S1(i,j)+(1-\eta )\times S2(i,j)$$

Parameter $\eta \in [0,1]$ is the integrated parameter and $FS(i,j)\in [0,1]$ is the final prediction score between lncRNA *i* to disease *j*.

## Electronic supplementary material

Supplementary file illustrate

Table s1

Table s2

Table s3
