# Supplementary material for: Global network random walk for predicting potential human lncRNA-disease associations
Source: Sci Rep. 2017 Sep 29;7:12442. doi: 10.1038/s41598-017-12763-z (PMC5622075; doi:10.1038/s41598-017-12763-z)
Supplement: Supplementary file 1 — Supplementary file illustrate [file 41598_2017_12763_MOESM1_ESM.doc]

**Global network random walk for predicting potential human lncRNA-disease associations**

Changlong Gu^1^, Bo Liao^1,*^, Xiaoying Li^1^, Lijun Cai^1^, Zejun Li^1,2^, Keqin Li^3^ and Jialiang Yang^4^

^1^College of Information Science and Engineering, Hunan University, Changsha, Hunan 410082, China

^2^School of Computer and Information Science,Hunan Institute of Technology, Hengyang 412002,China

^3^Department of Computer Science, State University of New York, New Paltz, New York 12561, USA

^4^Department of Genetics and Genomic Sciences,Icahn School of Medicine at Mount Sinai, New York 10029, USA

*Corresponding authors

**Email:** [dragonbw@163.com](mailto:dragonbw@163.com)

**Supplementary data file**

**Tables Legends**

**Table s1:** The benchmark dataset. 210 distinct high-quality experimental verified lncRNA-disease associations are obtained, including 78 lncRNAs and 113 diseases.

**Table s2:** Disease semantic similarities of 113 diseases in our benchmark dataset.

**Table s3:** LncRNA functional similarities of 78 lncRNAs in our benchmark dataset.
